# Impact of Ultrasound Extraction Parameters on the Antioxidant Properties of *Moringa Oleifera* Leaves

**DOI:** 10.3390/antiox9040277

**Published:** 2020-03-26

**Authors:** Luna Pollini, Carmela Tringaniello, Federica Ianni, Francesca Blasi, Jordi Manes, Lina Cossignani

**Affiliations:** 1 Department of Pharmaceutical Sciences, University of Perugia, Via San Costanzo, 06126 Perugia, Italy; luna.pollini@studenti.unipg.it (L.P.); carmela.tringaniello@unipg.it (C.T.); federica.ianni@unipg.it (F.I.); lina.cossignani@unipg.it (L.C.); 2 Laboratory of Food Chemistry and Toxicology, Faculty of Pharmacy, University of Valencia, Av. Vicent Andrés Estellés s/n, 46100 Burjassot, Spain; Jordi.Manes@uv.es

**Keywords:** *Moringa oleifera* leaves, UAE, phenol compounds, optimization, liquid chromatography

## Abstract

Recently, much interest has been focused on *Moringa oleifera* L., a highly versatile and sustainable plant. In addition to its nutritional properties, numerous bioactive compounds have been identified in *M. oleifera* leaves, for which healthy properties have been reported. In the present research, the impact of ultrasound-assisted extraction (UAE) on the recovery of the bioactive compounds from leaves was investigated. Firstly, an experimental design approach has been used to highlight the influence of some extraction parameters (solvent, solvent/dry leaves ratio, temperature, time) on phenol compound recovery and antioxidant activity. Solvent composition was the most influential factor; in fact, the presence of water in the solvent (50:50, v/v) corresponded to an increase in the extraction performance. The liquid/solid ratio (L/S) also influenced the extraction process; in fact, the total phenol content reached 13.4 mg gallic acid equivalent (GAE)/g dry matter (DM) in the following UAE conditions: 50% water, 60:1 L/S ratio, 60 °C, 60 min. In order to quantify flavonols, hydroalcoholic extracts were analysed by HPLC-DAD (high performance liquid chromatography-diode array detector). In the flavonol class, the glycosidic forms of quercetin and kaempferol were mainly detected. Their content ranged from 216.4 µg/g DM of quercetin 3-O-rhamnoside to 293.9 µg/g DM of quercetin 3-O-(6″-O-malonyl)-β-D-glucoside. In summary, the leaves of *M. oleifera* are a potential natural source of bioactive compounds, proving to be very promising for the development of health-promoting food supplements.

## 1. Introduction

Consumer awareness of the nutritional and health-promoting effects of food is constantly increasing, particularly in potential antioxidant compounds in addition to classical basic nutrients. Research into antioxidant sources is justifiable because there is increasing scientific evidence that various diseases are linked to oxidative stress [1,2].

In order to recover bioactive compounds with high efficiency, conventional extraction methods are used, among which maceration and percolation are the most popular, but large solvent amounts and long extraction times are required [3].

In recent years, in order to reduce these disadvantages, many advanced extraction methods have been developed. The recovery of bioactive compounds by these extraction techniques is a promising trend in the field of nutraceuticals and functional food development. Among the non-conventional techniques, subcritical extraction, pressurized liquid extraction, microwave-assisted extraction (MAE) and ultrasound-assisted extraction (UAE) have been studied for the extraction of phenol compounds from *Moringa oleifera* L. [4,5,6,7].

Due to the high extraction efficiency and popularity of the ultrasonic equipment, UAE can be considered one of the most practical extraction methods for recovering bioactive compounds from plant materials, i.e., flowers, fruits, leaves, bark, seeds, and pods [3,8]. UAE can increase the rate of mass transfer of the extraction based on the cavitation generated within the material. The cell wall polymeric structure is disassembled, thereby enhancing the release of the bioactive compounds from plant material into the liquid extraction phase [9]. UAE allows for short extraction times due to the increase of the analyte’s solubility in the extraction media when surface tension and solvent viscosity decrease, improving the extraction efficiency. However, in large industrial or semi-industrial scales, even though UAE requires expensive equipment and demands high energy consumption, it has been successfully used with improved bioactive compound yields for the extraction of various foods and waste, including *Moringa oleifera* leaves [7,10,11].

*Moringa oleifera* Lam. is the most widely cultivated species of a monogeneric family, the Moringaceae, native to the sub-Himalayan regions [12]. Despite its ancestral usefulness, its application has been rather empirical and most of the existing information about it comes from oral tradition [13]. Only at the end of the 20th century did this tree receive deserved attention from the scientific community; in fact, numerous reports have been published on the chemical composition and nutritional properties of the different organs of the plant [12,14,15,16], as well as the identification of bioactive compounds and their mechanisms of action [17,18,19]. In oil processing, the seed pods from *M. oleifera* are used to obtain commercial products such as Ben oil, while other organs of the tree, such as the leaves, are discarded as waste. The leaves have traditionally been used in Chinese medicine due to their beneficial bioactivities for human health [14], and numerous recent studies confirm these health positive effects, such as antimicrobial, anticancer, antiulcer, analgesic, and antihypertensive properties. Recently, some authors have reviewed animal and human studies carried out on *M. oleifera* leaves to evaluate glycaemia and insulin levels [20].

The purpose of this study was to optimize the ultrasonic extraction of bioactive compounds by response surface methodology (RSM). Four parameters of extraction were investigated: solvent, liquid/solid ratio, time, and temperature. In order to evaluate the recovery of bioactive compounds, the following responses were considered: total phenol content, total flavonoid content and antioxidant capacity measured by DPPH (2,2-diphenyl-1-picrylhydrazyl) and FRAP (ferric-reducing antioxidant power) in vitro assays. In addition, the extract obtained in the optimized conditions was analyzed by HPLC-DAD (high performance liquid chromatography-diode array detector) and UHPLC-MS/MS (ultra-high performance liquid chromatography-tandem mass spectrometry) in order to characterize the qualitative and quantitative profile of the flavonol fraction.

## 2. Materials and Methods

### 2.1. Plant Material and Chemicals

Dried leaves of *Moringa oleifera* L. were acquired from a herbalist’s shop. On the label, Italian origin (Salento, South Italy) was declared. Gallic acid, 2,2-diphenyl-1-picrylhydrazyl (DPPH radical), 2,4,6-tripyridyl-s-triazine (TPTZ), sodium carbonate (Na_2_CO_3_), aluminium chloride (AlCl_3_), iron(III) chloride hexahydrate (FeCl_3_·6H_2_O), Folin and Ciocalteu’s phenol reagent, (±)-6-hydroxy-2,5,7,8-tetramethylchromane-2-carboxylic acid (Trolox), kaempferol (≥90%), quercetin (≥95%), kaempferol-3-O-glucoside (≥95%), kaempferol-3-O-D-galactoside (≥90%), quercetin-3-D-galactoside (≥97.0%), quercetin-3-O-rhamnoside (≥78%), quercetin-3-β-D-glucoside (≥90%), quercetin 3-O-(6″-O-malonyl)-β-D-glucoside (≥85%) were from Sigma–Aldrich (Milan, Italy). HPLC grade and analytical grade solvents were acquired from VWR (Milan, Italy). Deionized water was obtained using a Milli-Q system (Millipore Corp, Billerica, MA, America).

### 2.2. Optimization of Phenol Extraction from Moringa Leaves by UAE

The extractions were carried out in an ultrasonic bath (AU-65; ArgoLab, Carpi, Italy), consisting of a stainless steel jug with a maximum capacity of 6500 mL. The influence of some parameters on the efficiency of the extraction of bioactive compounds from *M. oleifera* leaves and the optimization of phenol extraction conditions have been studied using the Statistical Design Package MODDE 5.0^TM^, an experimental design software (UMETRICS, Umeå, Sweden).

The following factors have been considered:

✓ solvent composition: quantitative multilevel factor, with H_2_O % in methanol, setting at values of 0 and 50;
✓ liquid/solid ratio, i.e., liquid/dry leaves, (indicated as L/S): quantitative multilevel factor, with values set at 30 and 60, expressed as liquid volume/leaf weight in grams of dry matter (mL/g DM);
✓ time: quantitative factor, with values set from 10 to 60 min;
✓ temperature: quantitative factor, with values set from 30 to 60 °C.

The following responses have been selected:

✓ total phenol content (TPC), expressed as milligrams of gallic acid equivalent per gram of dry matter (mg GAE/g DM);
✓ total flavonoid content (TFC), expressed as milligrams of quercetin equivalent per gram of dry matter (mg QE/g DM);
✓ free radical-scavenging activity using DPPH, expressed as percentage of antioxidant activity (AA%);
✓ ferric reducing antioxidant power (FRAP) assay, expressed in µmol Fe^+2^ per gram of dry matter (µmol Fe^+2^/g DM).

Factors and responses of the experimental design are summarized in Table 1 and Table 2, together with unit, type and setting values for factors, and the unit and acronym for responses.

A fractional factorial design of resolution IV, a linear model, was employed and a total of 11 experiments, named N1 to N11, was obtained (including three replicated centre points). The extractions were carried out in random order using the experimental conditions indicated in the worksheet (Table 3). The model was then fitted using the partial least squares (PLS) regression analysis.

### 2.3. Determination of Total Phenol Content

The total phenol content (TPC) was determined by a spectrophotometric method, according to the Folin–Ciocalteu procedure, modified by Pagano et al. [21]. Deionized water, 20% Na_2_CO_3_ solution and Folin and Ciocalteu’s reagent were added to an aliquot of the diluted extract. The solution was left to react in the dark for 30 min, after which the absorbance at 750 nm was measured using a Lambda 20 spectrophotometer (PerkinElmer, Inc; Waltham, Massachusetts, USA). The results were expressed as mg GAE/g DM, using a gallic acid calibration curve.

### 2.4. Determination of Total Flavonoid Content

The total flavonoid content (TFC) was determined by a spectrophotometric method [22]. Methanol, 10% AlCl_3_, 1 M potassium acetate and H_2_O were added to an aliquot of the appropriate diluted extract. The mixture was incubated in the dark for 30 min, after which the absorbance at 415 nm was measured. The results were expressed as mg QE/g DM, using a quercetin calibration curve.

### 2.5. In Vitro Antioxidant Activities

#### 2.5.1. Free Radical-Scavenging Activity using DPPH (DPPH Assay)

A DPPH assay was carried out according to the procedure reported by Blasi et al. [23]. DPPH (0.06 mM in ethanol) was added to the sample and the mixture was kept in the dark for 30 min, after which the absorbance at 517 nm was measured.

The percentage of antioxidant activity (AA%) for each sample was calculated using the following formula:

$$DPPH\;\left(AA\;\%\right)=\frac{\left(Absc-Abss\right)}{Absc}\;\times \;100$$

where Absc is the absorbance of the control solution containing only DPPH radical and Abss is the absorbance of the DPPH solution containing the sample.

#### 2.5.2. Ferric Reducing Antioxidant Power (FRAP) Assay

The reducing capacity of the leaf extracts was determined according to the procedure reported by Moscatello et al. [24]. The FRAP reagent, prepared by mixing acetate buffer, TPTZ and FeCl_3_·6H_2_O, was added to the leaf extracts, and then the samples were left in the dark for 30 min. The absorbance of the sample was measured at 593 nm. The results were expressed as µmol Fe^+2^/g DM, using a calibration curve prepared with solutions of known Fe^+2^ concentrations.

### 2.6. Analysis of Flavonols by HPLC-DAD

The HPLC analysis was performed using a Thermo Spectraseries HPLC, coupled with the Spectra System UV6000LP DAD (Thermo Separation Products, San Jose, CA, USA), according to a previous paper [25]. The chromatographic separation of phenol compounds was carried out with a Hypersil GOLD column (150 mm × 4.6 mm, 3 µm particle size). Separation was achieved using a gradient elution of solvent A (0.1% formic acid in water) and solvent B (0.1% formic acid in acetonitrile): B increased from 5% to 20% in 30 min, and then to 95% in 5 min (flow: 1 mL/min). Standard solutions containing phenol compounds (kaempferol, quercetin, kaempferol-3-O-glucoside, kaempferol-3-O-D-galactoside, quercetin-3-D-galactoside, quercetin-3-O-rhamnoside, quercetin-3-β-D-glucoside, and quercetin 3-O-(6″-O-malonyl)-β-D-glucoside) were used to identify and quantify the analytes. Peak identification was confirmed by UHPLC-MS/MS analysis [26].

### 2.7. Statistical Analysis

The results of HPLC analysis were expressed as the mean and standard deviation, based on three replicates. Microsoft Excel 2013 (Microsoft Corporation, Redmond, WA, USA) was used for data analysis.

## 3. Results and Discussion

### 3.1. Optimization of Extraction Conditions of Phenols from Moringa Leaves by UAE

The optimization study was performed by experimental design, a technique for planning experiments, that allows us to use a minimum number of experiments in which several experimental parameters vary simultaneously. Based on the obtained data, a mathematical model of the studied process is created. The model can be used to understand the influence of the experimental parameters on the response and to find the optimal conditions for the process [27]. Regardless of application domain, this methodology is useful for three objectives: screening, optimization, and robustness testing. Employed at the beginning of the investigation of a new application, screening experiments are commonly designed to explore many factors in order to evaluate their effects on the responses. Response surface methodology (RSM) is an approach based on mathematical and statistical techniques that is useful in designing experiments and evaluating the effects of factors. RSM, a useful tool to build empirical models and to determine the optimum conditions for a desirable response, has been widely used for various process optimizations [28,29], including the extraction of phenol from plants [7,23,30].

To evaluate the influence of UAE conditions of *Moringa* leaves on total phenol content (TPC), total flavonoid content (TFC) and antioxidant activity (FRAP, DPPH), the software MODDE 5.0 ^TM^ was used. In this work, pure methanol and hydroalcoholic solution (methanol/water 50:50, v/v) were used as extraction solvents. The solvent/dry leaves ratio (mL/g DM), indicated as the liquid/solid ratio (L/S), is also an influential parameter on extraction results and the ratios of 60:1 and 30:1 have been considered. Other important parameters affecting the extraction results were considered, such as time (ranging from 10 to 60 min) and temperature (ranging from 30 and 60 °C). Selecting the screening objective and a fractional factorial design of IV resolution, the worksheet reported in the Table 3 was obtained. The experiments N1–N11 have been carried out, and the TPC, TFC and antioxidant activity (DPPH and FRAP) have been determined. The obtained results are shown in Table 4.

### 3.2. Model Statistics

Table 5 shows the coefficients and the relative standard errors and p-values for TPC, TFC, FRAP and DPPH responses. It is possible to observe that the solvent and L/S ratio variables were significant for all the considered responses. Moreover, temperature was also significant for TPC and FRAP responses while time was variable for DPPH.

The quality of the obtained mathematical model can be evaluated by two statistical criteria, goodness of fit (R^2^) and goodness of predictability (Q^2^). The first describes how well the model fits the experimental data, while the second describes how well the model will predict new data. When R^2^ and Q^2^ values are close to the unit, the model is considered a good model and it can be used for optimization and prediction [27]. Table 6 shows the R^2^ and Q^2^ values of the statistical models obtained with the four responses from the UAE experiments. It is possible to observe that all values are sufficiently high (R^2^ values were always higher than 0.897, while Q^2^ values were always higher than 0.706), and for this reason they indicate the goodness-of-fit of the obtained statistical models.

**Figure 1:**
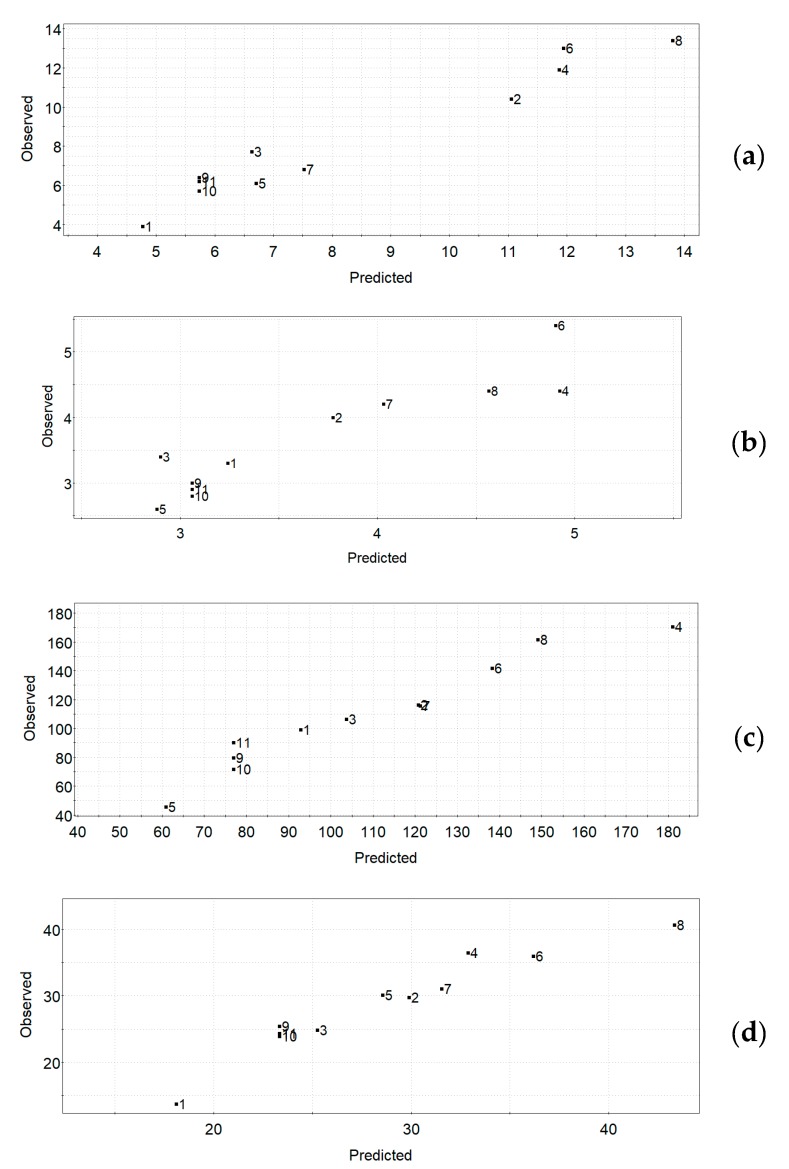
UAE experimental design. Observed vs. predicted plots for the responses: (**a**) TPC, (**b**) TFC, (**c**) FRAP, (**d**) DPPH.

Figure 1a–d shows the observed vs. predicted UAE plots for the four responses, respectively. The observed vs. predicted plot for a response can be used for the estimation of the quality of a model—for a good model, all the data points will fall on a straight line. The obtained plots indicate quite good models for all responses in the UAE experimental design.

### 3.3. Factor Influence on Responses

**Figure 2:**
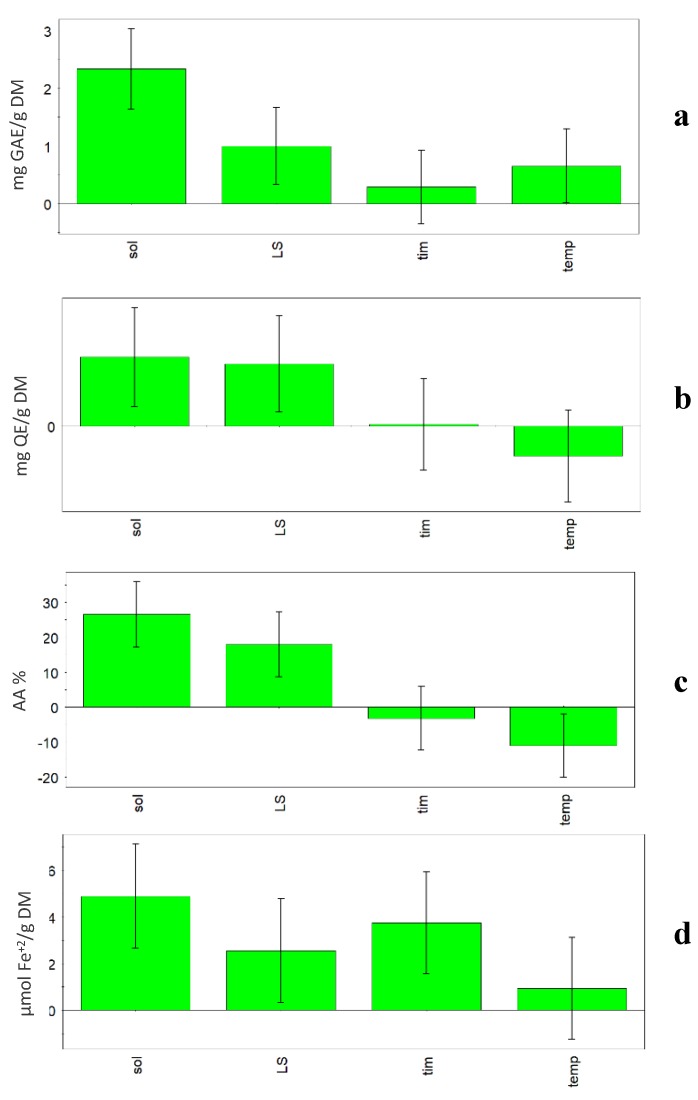
UAE experimental design. Coefficient plots showing the effect of solvent (sol), liquid/solid ratio (L/S), time (tim) and temperature (temp) on the responses: (**a**) TPC, (**b**) TFC, (**c**) FRAP, (**d**) DPPH.

Previous research carried out on vegetable sources has shown that factors such as solvent properties and volume, extraction time and temperature, and frequency and power of the ultrasound apparatus influence the extraction efficiency, and subsequently have an impact on the bioactivity of the extract [3,9,15]. In this research, when the model was built, some relevant factors (%H_2_O, L/S ratio, temperature, and time) were investigated and in Figure 2, the coefficient plots show the effect of the considered factors on the four responses (TPC, TFC, FRAP and DPPH). In all plots, it is evident that the solvent composition is the most influential factor among the ones considered. In fact, all four considered responses increased when the percentage of water increased in the solvent (50% v/v). Generally, hydroalcoholic extracts had higher TPC values than methanolic ones (*p* < 0.01), independently from the other variables. Among extraction conditions, the solvent has great influence on the extract composition and antioxidant activity, as reported by various authors [4,30].

Also, TFC content was significantly higher in hydroalcoholic than methanolic extracts (*p* < 0.05). L/S ratio was also an influential factor, but only in the case of TFC were the bars of solvent and L/S ratio comparable, so the two parameters showed a comparable influence on the extraction.

A positive influence on the extraction results (TPC and DPPH) was also obtained for the factors time and temperature. The statistical models showed that high time (60 min) and temperature (60 °C) during extraction lead to higher TPC and DPPH values. In fact, the Optimizer function (criterion Maximize) gave the best value of TPC (13.79 mg GAE/g DM) and DPPH (43.32%) using the following conditions: 50% H_2_O, 60:1 L/S ratio, 60 min and 60 °C. On the other hand, it must be taken into account that high temperatures could lead to negative changes for compounds in the considered matrix.

Generally, time has very little influence on the responses. In fact, with the exception of DPPH, the coefficient is slightly positive for TPC, slightly negative for FRAP and almost null for TFC. Temperature showed a negative effect on TFC and FRAP responses. In fact, the Optimizer function (criterion Maximize) gave the best value of TFC (4.75 mg QE/g DM) using the following conditions: 50% H_2_O, 60:1 L/S ratio, 32 min and 30 °C, while the best value of FRAP (180.99 µmol Fe^+2^/g DM) was obtained using the following conditions: 50% H_2_O, 1:60 L/S ratio, 10 min and 30 °C. 

Zhao et al. [7] reported that the best extraction conditions were ethanol 70%, 1:30 solid/liquid ratio, 50 °C temperature and 42 min time when using ultrasonic circulating extraction equipment at 300 W. Different optimal conditions (1:52 solid/liquid ratio, 43 min and 76 °C) were obtained for maximizing the extraction of flavonoids from *M. oleifera* using an ultrasonic bath cleaner at 40 KHz and 300 W [10].

**Figure 3:**
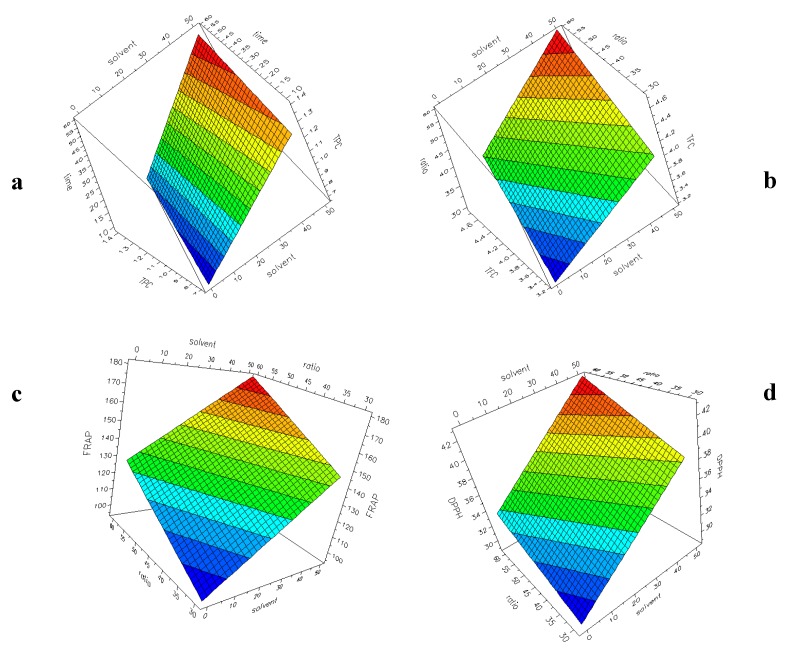
UAE experimental design. Surface plot showing the responses: (**a**) TPC (60:1, 60 °C); (**b**) TFC (10 min, 30 °C); (**c**) FRAP (10 min, 30 °C); (**d**) DPPH (60 min, 60 °C).

Figure 3a–d shows the surface plots with the four responses (TPC, TFC, FRAP, DPPH) as a function of two selected factors for UAE experiments. The plots have been generated by the software, setting a constant value for the other factors. The response–surface plot is generated to obtain a graphical representation of the experimental region. From this plot, the most interesting area can be used to verify experiments and to plan new experiments. The colour changing from blue to red indicates an increase in the response. It is evident that in all the considered models, an increase of H_2_O% into the solvent and a higher L/S ratio (60:1) correspond to an increase in the responses, as already highlighted during the discussion of the coefficients reported in Figure 2a–d.

### 3.4. Comparison with Literature Data (TPC and TFC)

It is known that differences in TPC, TFC and antioxidant activity in plant materials depend on cultivar, growing environment, extraction method, and so on [15,23].

Based on the condition of UAE extraction, *M. oleifera* leaves showed TPC values ranging from 3.9 to 13.4 mg GAE/g DW (Table 4; N1 and N8, respectively). The highest TPC value was obtained using the following conditions: hydroalcoholic solvent, 60:1 ratio, 60 min and 60 °C. Generally, the samples obtained with 50% H_2_O showed higher TPC values (10.4–13.4 mg GAE/g DW) than those obtained with pure MeOH (3.9–7.7 mg GAE/g DW). Other authors have studied the influence of the extraction solvent on TPC yield from *M. oleifera* leaves. For example, Rodríguez-Pérez et al. [30] have reported that the presence of water in the extraction solvent gave higher TPC results, with values of 46 mg GAE/g DW using MeOH:H_2_O (50:50) and 23.2 mg GAE/g DW using MeOH, for samples collected in Madagascar. However, it is important to observe that the extracts were first obtained by maceration and then by four successive UAE extractions. The same authors [5] have optimized the MAE extraction conditions to obtain phenol compounds from the same *M. oleifera* leaves and reported higher TPC values with a 50% hydroethanolic mixture (105.70 mg GAE/g DM) than those reported for ethanolic extracts (23.69 mg GAE/g DM). It should be noted that the cited results have been obtained by MAE extraction at 180 °C.

TPC values similar to those obtained in this research (Table 4) were reported by Castro-Lopez et al. [15] for *M. oleifera* leaves collected in Mexico. They used UAE with deionized water (40 kHz; 60 min; 25 °C) and obtained about 3 mg GAE/g DW with a 1:25 solid/liquid ratio and about 12 mg GAE/g DW with a 1:50 solid/liquid ratio.

Generally, the TPC values of *M. oleifera* leaves reported in other studies are higher, but in many cases, it is difficult to carry out real comparisons because of the very different conditions or methods used.

The TPC value of *M. oleifera* leaves collected from Greece was 29.04 mg GAE/g DM with UAE, while TPC values ranged from 24.72 to 40.24 mg GAE/g DM when a pulsed electric field at room temperature was applied for 40 min [31].

Siddhuraju and Becker [32] studied *Moringa* leaf extracts from different geographic origins. They reported values ranging from 7.43 to 12.33 g GAE/100 g DM (Nicaragua), 5.25 to 8.87 g GAE/100 g DM (India), and 6.83 to 9.76 g GAE/100 g DM (Niger), using an apparatus consisting of a round-bottom flask with an attached reflux condenser. The lower values were obtained using water extracts, while the higher ones were obtained using 80% methanol extracts.

In this work, *M. oleifera* leaves showed TFC content ranging from 2.6 to 5.4 mg QE/g DM based on different UAE conditions (Table 4; N5 and N6, respectively). For both experiments, time and L/S ratio were the same (60 min and 1:30), but %H_2_O and temperature were different (alcoholic solvent and 60 °C for N5, hydroalcoholic solvent and 30 °C for N6). It should be noted that a good correlation was found between TPC and TFC values (R^2^ = 0.6740).

In the literature, a wide range of TFC values has been reported: 10.14–14.07 g of rutin equivalent (RE)/g DM for *M. oleifera* leaves from Central America, 3.26–5.92 g RE/g DM for samples from South Asia, and 7.32–10.19 g RE/g DM for samples from West Africa. Higher values were reported for samples from Egypt (47.04–62.53 mg QE/g DM) obtained by UAE extraction and purification of flavonoid compounds by macroporous resin [10]. The highest value of TFC was 192.36 mg RE/g DM for young leaves of *M. oleifera* from Kenya, firstly extracted with 90% ethanol for 3 h, and then re-extracted in an ultrasonic bath (200 W, 40 KHz) for 30 min [11].

### 3.5. Comparison with Literature Data (Antioxidant Activity)

The interest in *Moringa* leaves is due also to their antioxidant properties [6,7]. Therefore, in this work, the antioxidant activity of *Moringa* leaves by two in vitro complementary tests, named FRAP and DPPH, was investigated. Two tests were used as only one antioxidant mechanism did not give an overview of the antioxidant potential of the bioactive compounds, hence reducing the power, and the radical inhibiting property was analysed.

The FRAP method is a simple and effective procedure based on the reduction of a ferric tripyridyltriazine complex to its ferrous, coloured form in the presence of an antioxidant. The FRAP assay directly measures the ability of antioxidants to act as reducing compounds, showing a reduction potential below the reduction potential of the Fe^3+^/Fe^2+^ couple. *M. oleifera* leaves showed FRAP values ranging from 45.5 to 170.5 µmol Fe^+2^/g DM (Table 4) based on the different UAE conditions (N5 and N4, respectively). As already discussed for TPC, the hydroalcoholic extracts showed the highest values (from 116.4 to 170.5 µmol Fe^+2^/g DM) in respect to extractions carried out with pure MeOH (79.7–115.7 µmol Fe^+2^/g DM).

In regards to DPPH, the values ranged between 13.7% for N1 and 40.6% for N8 (Table 4); the N1 and N8 extracts were produced with very different extraction conditions (methanol, 30:1, 10 min, 30 °C vs. hydroalcoholic solvent, 60:1, 60 min, 60 °C, respectively).

A correlation study showed acceptable results between TPC and DPPH values (R^2^ = 0.7709), TPC and FRAP values (R^2^ = 0.6749), and between TFC and FRAP values (R^2^ = 0.7411).

Castro-Lopez et al. [15] reported values of 0.70 and 2.43 mg GAE/g DM for FRAP and values of 1.87 and 5.03 mg GAE/g DM for DPPH when using UAE with a 1:25 or 1:50 solid/liquid ratio, respectively. The same authors used other extraction methods (maceration, decoction, and MAE) and also found that a 1:50 ratio gave higher FRAP and DPPH values with respect to the 1:25 ratio. Xu et al. [11] studied the antioxidant activities of the crude extracts of *M. oleifera* and found a half maximal inhibitory concentration (IC_50_) value of 1.02 mg/mL for DPPH assay, and a value of 0.99 mM Fe^2+^/g for FRAP assay.

Bozinou et al. [31] obtained a FRAP value of 71.68 µmoL ascorbic acid equivalent/g DM and 64.82 %AA (DPPH assay) for extracts from *M. oleifera* from central Greece obtained with UAE at 36 °C for 15 min.

### 3.6. Characterization of the M. oleifera Leaf Extract: Flavonols

There is long-standing knowledge regarding phytochemicals present in different organs of *M. oleifera*, but only recently, Lin et al. [18] reviewed the current studies of the health-promoting aspects of *Moringa* flavonoids on cancer, diabetes and obesity. It has been reported that flavonols are the most common flavonoids found in *Moringa*. They are in abundance and linked to a wide spectrum of sugar moieties (e.g., acetyldihexose and hexose) and the glycon structures might greatly modulate the bioactivities of flavonoids [18]. Obviously, differences in the contents of bioactive compounds are most likely due to environmental conditions, geographic origin and other factors [32,33,34,35]. In this paper, the characterization of the flavonol fraction of *Moringa* leaves produced in Italy was carried out. Based on the results of optimization step, *M. oleifera* leaves have been extracted under the following experimental conditions: hydroalcoholic solvent, 60:1 L/S ratio, 35 min and 45 °C. Table 7 shows the main flavonols identified and quantified in the UAE extract of *M. oleifera* leaves. In Figure S1 the relative HPLC-DAD chromatogram is reported.

Various glycosidic forms of quercetin were identified in *M. oleifera* leaves [11,33], but the aglyconic forms obtained after acid hydrolysis were also reported [32,36]. For example, quercetin and kaempferol contents changed respectively from 657 to 2749 mg/100 g DM and from 154 to 647 mg/100 g DM, considering samples from different origin (India, Niger and Nicaragua) and different extraction solvent [32].

In this research, kaempferol and quercetin (30.1 and 65.4 µg/g DM, respectively) are minor compounds, followed by the quercetin glycosidic forms, ranging from 216.4 µg/g DM of quercetin 3-O-rhamnoside to 293.9 µg/g DM of quercetin 3-O-(6″-O-malonyl)-β-D-glucoside. 

At least fourteen flavonoids were identified by UHPLC analysis coupled with qTOF-MS (quadrupole time-of-flight mass spectrometry) in *M. oleifera* leaves, harvested in Namibia and South Africa [33]. Coppin et al. [36] identified twelve flavonoids (six kaempferol derivatives and six quercetin derivatives) in *M. oleifera* leaves collected from sub-Saharan Africa using HPLC coupled with UV and mass spectrometer detection systems; the total amount of flavonoids (quercetin plus kaempferol) ranged from 0.18 g/100 g DM for indigenous Dakar samples to 1.64 g/100 g DM for *M. oleifera* leaves originally developed in India and commercially available. Xu et al. [11] identified five flavonoids (rutin, quercetin 3-O-glucoside, quercetin-acetyl-glycoside, kaempferol 3-O-glucoside, and kaempferol-acetyl-glycoside) from leaves, but also from root and seeds, of *M. oleifera* collected in Kenya using LC-MS/MS.

## 4. Conclusions

In this study, an experimental design approach was successfully applied for investigating the effect of some extraction factors on UAE extraction of bioactive compounds with antioxidant activity from *M. oleifera* leaves. UAE technique, minimizing process time, and temperature are useful for the extraction of thermolabile compounds, such as phenol compounds. For these reasons, this technique represents a sustainable alternative for the use of agro-industrial residues through the efficient extraction of bioactive compounds.

The influence of some extraction parameters on phenol and flavonoid contents and antioxidant properties was studied. The results showed that the composition of the extraction solvent and the liquid/solid ratio were the most significant factors affecting the extraction of bioactive compounds from *M. oleifera* using the UAE process. The best UAE conditions were used to extract phenols from a commercial sample of *Moringa* leaves. Then, the flavonol fraction was characterized by HPLC-DAD and UHPLC-MS/MS. In conclusion, the results of this study show that the optimized non-conventional extraction technique is a simple and efficient alternative for the recovery of flavonoids from *Moringa* leaves, a promising source of bioactive compounds for functional food and nutraceutical development. Future research directions could be aim towards the purification and isolation of these bioactive compounds, and whether this process could be exploited on an industrial scale.

## Figures and Tables

**Table 1:** Factors set in the ultrasound-assisted extraction (UAE) experimental design.

Factor	Unit	Type	Setting
Solvent	H_2_O %	quantitative multilevel	0, 50
Liquid/solid ratio	mL/g DM	quantitative multilevel	30, 60
Time	min	multilevel	10 to 60
Temperature	°C	quantitative	30 to 60

**Table 2:** Responses set in the UAE experimental design.

Response	Unit	Acronym
Total phenol content	mg GAE/g DM	TPC
Total flavonoid content	mg QE/g DM	TFC
Free radical-scavenging activity	AA%	DPPH
Ferric reducing antioxidant power	µmol Fe^+2^/g DM	FRAP

GAE = gallic acid equivalent; QE = quercetin equivalent; DM = dry matter; AA% = % of antioxidant activity

**Table 3:** Worksheet with experimental conditions for UAE.

	Solvent	L/S Ratio	Time	Temperature
	H_2_O %	mL/g DM	min	°C
N1	0	30	10	30
N2	50	30	10	60
N3	0	60	10	60
N4	50	60	10	30
N5	0	30	60	60
N6	50	30	60	30
N7	0	60	60	30
N8	50	60	60	60
N9	0	30	35	45
N10	0	30	35	45
N11	0	30	35	45

**Table 4:** Responses for fractional factorial IV resolution design.

	TPC	TFC	FRAP	DPPH
	mg GAE/g DM	mg QE/g DM	µmol Fe^+2^/g DM	AA%
N1	3.9	3.3	99.2	13.7
N2	10.4	4.0	116.4	29.7
N3	7.7	3.4	106.5	24.8
N4	11.9	4.4	170.5	36.4
N5	6.1	2.6	45.5	30.1
N6	13.0	5.4	141.6	35.9
N7	6.8	4.2	115.7	31.0
N8	13.4	4.4	161.5	40.6
N9	6.4	3.0	79.7	25.4
N10	5.7	2.8	71.6	23.9
N11	6.2	2.9	90.3	24.3

Abbreviations are described in Table 1 and Table 2.

**Table 5:** Model coefficients (Coeff.), standard error (Std. Err.) and p-values for the considered responses.

	**TPC**			**TFC**		
	Coeff.	Std. Err.	p-value	Coeff.	Std. Err.	p-value
constant	7.8500	0.2221	**3.41** **×** **10^−7^**	3.5000	0.0944	**2.69** **×** **10^−7^**
solvent	2.3366	0.2692	**0.0003**	0.4104	0.1144	**0.0157**
L/S ratio	1.0009	0.2611	**0.0122**	0.3703	0.1110	**0.0206**
time	0.2897	0.2493	**0.2976**	0.0114	0.1059	**0.9179**
temperature	0.6537	0.2493	**0.0469**	-0.1783	0.1059	**0.1532**
	**DPPH**				**FRAP**	
	Coeff.	Std. Err.	p-value	Coeff.	Std. Err.	p-value
constant	108.9550	3.5390	**7.80** **×** **10^−8^**	28.7091	0.8509	**4.51** **×** **10^−8^**
solvent	4.8941	0.9137	**0.0017**	26.5327	3.8001	**0.0004**
L/S ratio	2.5518	0.9137	**0.0314**	17.9207	3.8001	**0.0032**
time	3.7401	0.8925	**0.0057**	-3.22185	3.7118	**0.4187**
temperature	0.9293	0.8925	**0.3378**	-11.0545	3.7118	**0.0246**

Significant p-values (≤ 0.05) are identified in bold.

**Table 6:** Goodness of fit (R^2^) and goodness of predictability (Q^2^) coefficients of statistical models.

	R^2^	Q^2^
TPC	0.931	0.706
TFC	0.897	0.708
FRAP	0.943	0.795
DPPH	0.914	0.726

**Table 7:** Flavonol composition of *M. oleifera* leaves (mean value ± standard deviation, *n* = 3).

Compounds	UAE	
	%	µg/g DM
Quercetin 3-O-galactoside	17.1 ± 0.3	271.8 ± 6.7
Quercetin 3-O-glucoside	17.2 ± 0.3	272.8 ± 6.7
Quercetin 3-O-(6″-O-malonyl)-β-D-glucoside	18.5 ± 0.4	293.9 ± 4.9
Quercetin 3-O-rhamnoside	13.6 ± 0.2	216.4 ± 0.5
Kaempferol 3-O-galactoside	13.7 ± 0.1	217.4 ± 0.5
Kaempferol 3-O-glucoside	13.8 ± 0.1	218.4 ± 0.6
Quercetin	4.1 ± 0.1	65.4 ± 1.2
Kaempferol	1.9 ± 0.0	30.1 ± 0.3

## Supplementary Materials

The following are available online at https://www.mdpi.com/2076-3921/9/4/277/s1, Figure S1: HPLC-DAD separation of flavonols from Moringa leaves. The extract has been obtained using the following experimental conditions: hydroalcoholic solvent, 60:1 L/S ratio, 35 min and 45 °C. Peak assignments: 1. Quercetin 3-O-galactoside, 2. Quercetin 3-O-glucoside, 3. Quercetin 3-O-(6″-O-malonyl)-β-D-glucoside, 4. Quercetin 3-O-rhamnoside, 5. Kaempferol 3-O-galactoside, 6. Kaempferol 3-O-glucoside, 7. Quercetin, 8. Kaempferol title
