# Supplementary material for: Impact of Ultrasound Extraction Parameters on the Antioxidant Properties of Moringa Oleifera Leaves
Source: Antioxidants (Basel). 2020 Mar 26;9(4):277. doi: 10.3390/antiox9040277 (PMC7222185; doi:10.3390/antiox9040277)
Supplement: Supplementary file 1 [file antioxidants-09-00277-s001.pdf]

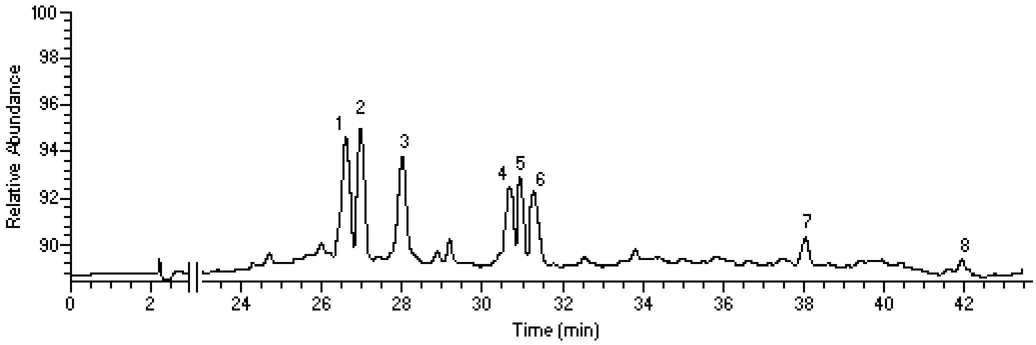

Figure S1. HPLC-DAD separation of flavonols from Moringa leaves. The extract has been obtained using the following experimental conditions: hydroalcoholic solvent, 60:1 L/S ratio, 35 min and 45 °C. Peak assignments: 1. Quercetin 3-O-galactoside, 2. Quercetin 3-O-glucoside, 3. Quercetin 3-O-(6”-O-malonyl)-β-D-glucoside, 4. Quercetin 3-O-rhamnoside, 5. Kaempferol 3-O-galactoside, 6. Kaempferol 3-O-glucoside, 7. Quercetin, 8. Kaempferol
